# Evaluation of Microglial Activation in Multiple Sclerosis Patients Using Positron Emission Tomography

**DOI:** 10.3389/fneur.2018.00181

**Published:** 2018-03-26

**Authors:** Laura Airas, Marjo Nylund, Eero Rissanen

**Affiliations:** ^1^Division of Clinical Neurosciences, Turku University Hospital and University of Turku, Turku, Finland; ^2^Turku PET Centre, Turku University Hospital and University of Turku, Turku, Finland

**Keywords:** microglia, positron emission tomography, imaging, 18-kDa translocator protein, multiple sclerosis

## Abstract

Understanding the mechanisms underlying progression in multiple sclerosis (MS) is one of the key elements contributing to the identification of appropriate therapeutic targets for this under-managed condition. In addition to plaque-related focal inflammatory pathology typical for relapsing remitting MS there are, in progressive MS, widespread diffuse alterations in brain areas outside the focal lesions. This diffuse pathology is tightly related to microglial activation and is co-localized with signs of neurodegeneration. Microglia are brain-resident cells of the innate immune system and overactivation of microglia is associated with several neurodegenerative diseases. Understanding the role of microglial activation in relation to developing neurodegeneration and disease progression may provide a key to developing therapies to target progressive MS. 18-kDa translocator protein (TSPO) is a mitochondrial molecule upregulated in microglia upon their activation. Positron emission tomography (PET) imaging using TSPO-binding radioligands provides a method to assess microglial activation in patients *in vivo*. In this *mini-review*, we summarize the current status of TSPO imaging in the field of MS. In addition, the review discusses new insights into the potential use of this method in treatment trials and in clinical assessment of progressive MS.

## Introduction

Multiple sclerosis (MS) is a chronic autoimmune disease of the central nervous system (CNS) which leads to demyelination and neurodegeneration. In 85% of cases, MS starts as a relapsing remitting disease following an attack against the CNS by the adaptive immune system. This leads to formation of MRI-detectable, gadolinium-enhancing focal inflammatory lesions. Depending on the anatomical location of the lesions, neurological symptoms, i.e., MS relapses, will follow. Inflammation within the CNS contributes to demyelination and neuronal damage (1). Within 10–15 years after the diagnosis, more than 60% of RRMS patients procede to develop secondary progressive MS (SPMS) in which relapses give way to relentless disease progression and accumulation of disability (2). This progression is associated with activation of the local innate immune system within the CNS and, gradually, white blood cell trafficking from the periphery into the CNS is reduced (3). Both resident microglia and blood-derived macrophages contribute to neuronal damage *via* release of pro-inflammatory cytokines and reactive oxygen species (4). These lead to oxidative injury of mitochondria and to oligodendrocyte damage and degeneration of neurons (5, 6). The resulting energy failure and membrane channel dysfunction may be key processes in progressive disease. Interfering with these mechanisms, for example by reducing the harmful pro-inflammatory microglia functions, may provide neuroprotection and prevent disability progression by myelin repair and restoration of axonal activity and conduction.

Neuropathological studies have demonstrated that MS lesions in progressive disease rarely have features of acute inflammation. Instead, brain samples from patients with progressive disease have chronic active (smoldering or expanding) lesions with microglial activation at the edge of an otherwise burned out plaque (7). Alternatively, the chronic lesions are inactive, with no microglial activation at the plaque edge (7). In addition, widespread microglial activation is seen in areas surrounding the focal lesions, in the so called normal-appearing white matter (NAWM) (8). Microglial activation is associated with signs of neuronal damage and tissue atrophy and hence it is assumed that microglial cells contribute to the CNS damage of progressive MS (9). In this narrative *mini-review*, we give a comprehensive overview of the present state of the use of positron emission tomography (PET) using 18-kDa translocator protein (TSPO)-binding radioligands for imaging of microglial activation in MS. We have used PubMed for literature searches using the following search terms: TSPO imaging, neuroinflammation, PET, and MS. We discuss the promise and potential of TSPO imaging in *in vivo* visualization of microglial activation in association with various aspects of MS, address significant gaps in the field and highlight future directions for further investigation.

## Why New Imaging Methods are Needed for the Study of MS?

Given the current limited understanding of the neuropathological process of progressive MS, it is not surprising that the disease modifying treatments used successfully to treat RRMS, which mostly function on the peripheral adaptive immune system, are not effective for progressive MS. Attempts to find treatments for progressive MS have proven challenging with, frequently, disappointing results (10). However, recently, ocrelizumab, a humanized monoclonal antibody selectively depleting CD20-expressing B-cells, was the first disease modifying treatment to show efficacy in slowing down disease progression in primary progressive MS (11). A breakthrough is still awaited for effective treatment of SPMS. Imaging methods or biomarkers for progressive MS, which would assist in treatment development, are not well established and the diagnosis is usually retrospective, based on the history of gradual neurological worsening with or without occasional relapses (12). Conventional MRI is sensitive in demonstrating the gadolinium enhancing active focal inflammatory lesions, and MRI is essential for MS diagnostics, clinical follow-up and treatment trials of RRMS. MRI studies in progressive MS, on the other hand, often demonstrate limited blood–brain-barrier (BBB) permeability. This is in accordance with the ongoing compartmentalized inflammation within the CNS which has been well demonstrated in progressive disease using neuropathological studies (13). Other MRI characteristics of progressive MS include increasing number and volume of T1-hypointense lesions, brain volume loss, changes in magnetic transfer imaging, and diffusion tensor imaging (14). Conventional MRI is not sensitive enough to visualize the diffuse pathology associated with progressive MS. Hence, more sensitive methods for monitoring progressive MS are urgently needed. PET imaging using radioligands binding to the TSPO molecule on activated microglial cells provides a method to specifically quantify microglial activation both in the context of the chronic lesions and within the NAWM. PET imaging will enable longitudinal *in vivo* follow-up of the pathobiology relevant to progressive MS, and it thus holds promise as a new outcome measure for treatment studies of this under-treated condition.

## Description of the PET Methodology

Positron emission tomography imaging uses short-lived radioactive isotopes bound to ligands that interact with their specific targets within the CNS (15). The radioactive isotopes emit positrons, that are detected using a sophisticated gamma-counter placed within a PET camera, and the amount of the bound ligand within the CNS can thus be quantitated. Radioligands used for PET imaging are produced by radiolabeling specific precursor molecules (the receptor ligands) with short-lived positron emitting isotopes, such as ^18^F and ^11^C using a cyclotron. Due to the short half-lives of the tracers, i.e., 20 min for a ^11^C-tracer or 110 min for a ^18^F-tracer, a short cyclotron-to-camera-time is required, and the radioligands must mostly be produced on-site. After an intravenous injection, the PET tracer enters the CNS, binds to its corresponding target and can be detected using the PET camera. PET imaging is a non-invasive imaging technique with high molecular sensitivity and specificity, which allows remarkably accurate *in vivo* quantification of the molecules of interest within the CNS (15–17). PET can be highly specific for a disease-related process, provided that a suitable PET tracer is available (18). PET imaging has been so far relatively underused in the evaluation of the disease pathogenesis in MS, despite the potential to be able to detect the pathogenic determinants related to MS pathogenesis *in vivo* and longitudinally in a given individual patient. Here, the detection of activated microglial cells in the context of progressive MS has been the main target of our PET imaging studies (19, 20).

## The TSPO-Molecule is Upregulated upon Activation of Microglia

For visualization of microglial activation, radioligands binding to the TSPO molecule are mostly used. TSPO is a protein structure, which is expressed on the outer mitochondrial membrane of activated microglia, and TSPO upregulation on microglial cells is thus considered to be a sensitive “real-time” marker of activation of these cells (21–23). TSPO is also expressed widely outside the CNS and it is thought to be involved in a range of vital cellular functions including regulation of cell proliferation, programmed cell death, steroid biosynthesis, and heme synthesis (24, 25). TSPO also plays a role in cell activation and in opening of the mitochondrial permeability transition pore (26). It was previously called the “peripheral benzodiazepine receptor” (27).

In the “resting” or surveying microglia, TSPO is expressed at a lower level; mainly in the gray matter (28). In non-neoplastic CNS damage without BBB breakdown, microglia are the main cell population expressing TSPO, but also blood-derived macrophages, reactive astrocytes, and endothelial and smooth muscle cells in the vasculature express TSPO (21, 29–33). Interestingly, knocking out TSPO is protective in a mouse model of MS (34). On the other hand, recent *in vitro* work investigated TSPO expression in activated macrophages and surprisingly, a consistent downregulation of TSPO mRNA and protein in macrophages activated to a pro-inflammatory, or “M1” phenotype was demonstrated (35). On the other hand, stimulation of macrophages to an M2 phenotype with IL-4, dexamethasone or TGF-β1 did not alter TSPO expression (35). The same group investigated TSPO expression in rodent vs. human-derived macrophages and microglia upon pro-inflammatory stimulation (36). Here, they demonstrated a ninefold increase in TSPO in rodent-derived macrophages and microglia upon pro-inflammatory stimulation, but surprisingly, TSPO expression did not increase with classical pro-inflammatory activation in primary human microglia. Pro-inflammatory activation of human monocyte-derived macrophages was associated with a reduction of both TSPO gene expression and TSPO-binding site availability. How these *in vitro* experiments relate to MS immunopathology in MS brain *in situ* remains to be seen, but the findings do suggest that changes in TSPO expression in PET imaging studies of MS may reflect microglial and macrophage density rather than activation phenotype (36). Neuropathological studies of TSPO localization in various types and various patho-anatomical locations in MS brain tissue *in situ* are, unfortunately, still relatively limited (21).

## Radioligands Used for Detection of TSPO

### First Generation TSPO Ligand [^11^C]PK11195

The first TSPO-binding compound, PK11195, has been available for more than 30 years (37). [^11^C]PK11195 was first used for imaging of human gliomas in 1989 (38), and the first *in vivo* human MS brain study was performed in 1997 (39). [^11^C]PK11195 has high specificity for TSPO (40), but a short half-life (20 min) and low signal-to-noise ratio complicates image analysis (41). [^11^C]PK11195, like other TSPO-ligands, binds to endothelial cells and to plasma proteins, which needs to be accounted for when evaluating the images. Quantification of specific radioligand binding in a given region of interest (ROI) usually requires comparison to a reference area devoid of specific binding. MS brain naturally lacks such an anatomically clearly defined reference region, which necessitates mathematical modeling of the signal to allow reliable estimation of specific binding to cells of the innate immune system (42–44). For quantification of specific [^11^C]PK11195 ligand binding, a semi-automated model (supervised clustering algorithm) has been validated (43, 44) and applied in several [^11^C]PK11195-PET studies of MS (20, 45, 46). Up to date, 12 different studies in MS using [^11^C]PK11195 have been published (Table 1). These studies have evaluated the presence of activated microglia in various cohorts of MS. They have also been used as a prognostic marker for worsening of the disease, or used for measuring the treatment effect of various MS treatments, as discussed below.

**Table 1:** Human *in vivo* positron emission tomography (PET) imaging studies with first generation TSPO ligand [^11^C]PK11195 in multiple sclerosis.

Reference	Study population (*n*)	Main findings on the radioligand binding		
		Lesion associated ROIs	NAWM/NAGM/other	Association with clinical parameters and/or with longitudinal outcome
Vowinckel et al. (39)	MS (2)	– Increased uptake in a resolving acute WM lesion – Low uptake in chronic T1 lesions	N/A	N/A

Banati et al. (30)	HC (8) RRMS (8) SPMS (1) PPMS (3)	– Higher uptake in 30% of Gd+ than Gd- lesions – Higher mean uptake in T1 black holes/hypointense lesions in RRMS patients during a relapse than without relapse – 1 SPMS patient with higher uptake in T1-hypointense lesions compared to RRMS	– Higher mean uptake in thalami and brainstem of MS vs. HC – Higher hemispheric percentage of voxels with increased (>2SD) binding in 4 patients compared to HC	– Association of higher percentage of TSPO-binding T1 lesion to higher EDSS

Debruyne et al. (47)	HC (7) RRMS (13) SPMS (7) PPMS (2)	– Increased uptake in Gd+ active lesions – Uptake in T2 lesions increased at the time of relapse	– No significant differences in NAWM and GM uptake between HC and all MS patients	– Higher NAWM uptake associated with longer disease duration

Versijpt et al. (48)	HC (8) RRMS (13) SPMS (7) PPMS (2)	– Lower uptake in T2 lesions associated with higher brain atrophy index^a^	– Higher uptake in NAWM associated with higher brain atrophy index^a^	N/A

Ratchford et al. (49)	RRMS (9)	N/A	– Decrease in global cortical GM and cerebral WM uptake after 1 year of treatment with glatiramer acetate	– Decrease in global cortical GM and cerebral WM uptake after 1 year of treatment with glatiramer acetate

Politis et al. (46)	HC (8) RRMS (10) SPMS (8)	N/A	– Higher cortical uptake in MS vs. HC and in wider areas in SPMS vs. RRMS – Higher uptake in WM of SPMS and RRMS vs. HC	– Total cortical binding correlated with EDSS, stronger association in SPMS than in RRMS – No association between Wm binding and clinical disability

Giannetti et al. (45)	RRMS (10) PMS (9; of which 8 SPMS, 1 PPMS)	– Heterogeneity in uptake within T1 black holes, 76% of black holes positive for [^11^C]PK11195 binding. No difference in distribution between RRMS and PMS – Uptake in [^11^C]PK11195 positive T1 black holes higher in PMS vs. RRMS	N/A	– Higher uptake in T1 black holes correlates with higher EDSS score in PMS but not in RRMS – Total binding in T1 black holes was a significant disability predictor in PMS at 2 years after TSPO-imaging

Rissanen et al. (20)	HC (8) SPMS (10)	– Increased perilesional uptake in 57% of T1-hypointense lesions – Mean uptake in T2 lesional area lower compared to NAWM in SPMS	– Higher uptake in NAWM and thalami in SPMS vs. HC	N/A

Giannetti et al. (50)	HC (8) CIS (18)	N/A	– Uptake in NAWM higher in CIS than in HC – Mean uptake in NAWM higher in patients with T2 lesions than without – Higher binding in deep but not in cortical GM in CIS vs. HC	– Higher uptake in NAWM correlated to higher EDSS – CIS subjects who developed CDMS by 2 years follow-up had higher uptake in NAWM at baseline

Tarkkonen et al. (51)	RRMS (1)	– Slightly but insignificantly increased [^11^C]PK11195 binding in a grade II glioma (astrocytoma) when compared to NAWM in a patient with RRMS	N/A	– Moderate uptake of [^11^C]methionine and non-specific uptake of [^11^C]PK11195. – Differentiation between a tumefactive demyelinating lesion and low-grade glioma not possible with PET in this case; biopsy confirmed the diagnosis

Sucksdorff et al. (52)	RRMS (11) HC (8)	– No significant difference in mean T2 lesional uptake compared to NAWM in baseline	– Higher uptake in combined NAWM+NAGM ROI and in thalami in RRMS vs. HC in baseline	– Decrease in mean T2 lesional uptake in group level after 6 mo. treatment with fingolimod

Kaunzner et al. (53)	RRMS (16) SPMS (2) HC (6)	– Significantly higher uptake in Gd+ and non-significant trend for higher uptake in Gd- lesions in MS patients compared to normal WM in HC at baseline	– No difference in cortical GM and thalamic binding among MS vs. HC at baseline – Good test-retest reproducibility in HC	– Decreased uptake in individual Gd+ lesions and decreased overall uptake in Gd- lesions at group level after 6 mo. treatment with natalizumab – No longitudinal changes in NAWM or NAGM

*TSPO, 18 kDa translocator protein; HC, healthy control; RRMS, relapsing remitting multiple sclerosis; SPMS, secondary progressive multiple sclerosis; PPMS, primary progressive multiple sclerosis; PMS, progressive multiple sclerosis; CIS, clinically isolated syndrome; ROI, region of interest; NAWM, normal-appearing white matter; WM, white matter; Gd+, gadolinium enhancing; Gd−, non-enhancing; EDSS, expanded disability status scale; CDMS, clinically definite MS*.

*^a^Brain atrophy index defined as the relative CSF volume divided by the relative white and gray matter volume*.

### Second-Generation TSPO Ligands

Second-generation TSPO ligands with higher affinity and specificity have been developed (23, 54), and over 80 high-affinity TSPO tracers are currently at some stage of development (55). Of these, [^11^C]PBR28, [^18^F]PBR111, [^11^C]FEDAA1106, and [^18^F]GE180 have already been used in studies of MS (56–60) (Table 2). The first studies with these tracers did not show differences in ligand uptake between MS patients and healthy controls (58, 61). However, this was before discovering that in humans the binding affinity for these second-generation ligands is individually determined by genetic variation in the TSPO gene. Thereafter, identification of a single nucleotide polymorphism (rs6971) in exon 4 of TSPO gene has enabled stratification of study subjects into high, medium, and low affinity binders (62), and thus, more accurate estimation of the ligand binding properties is possible at group level (Table 2).

**Table 2:** Human *in vivo* positron emission tomography (PET) imaging studies with second-generation TSPO ligands in multiple sclerosis.

TSPO ligand	Reference	Study population (*n*)	Main findings on the radioligand binding		
			Lesion associated ROIs	NAWM/NAGM/other	Association with clinical parameters and/or with longitudinal outcome
[^11^C]vinpocetine and [^11^C]PK11195	Vas et al. (63)^a^	MS (4; mainly^c^ RRMS)	– Lesional and perilesional binding of [^11^C]vinpocetine higher than with [^11^C]PK11195 but with low overlap in areas of high uptake between ligands	– Global uptake of [^11^C]vinpocetine higher than with [^11^C]PK11195	N/A

[^11^C]PBR28	Oh et al. (61)^a^	HC (7) MS (11; mainly^c^ RRMS)	– Increased uptake in Gd+ WM lesions – Varyingly increased perilesional binding in 71% of T1 lesions – Focally increased uptake in areas preceding development of Gd+ lesions^b^	– No difference in global uptake in MS vs. HC – Higher WM/GM binding ratio in MS vs. HC	– Correlation of higher global binding with longer disease duration, but not with EDSS or MSFC

	Park et al. (64)	HC (4) RRMS (4)	– No difference in T1 lesional vs. NAWM uptake in RRMS patients	– No differences in whole brain GM, whole brain NAWM or regional uptake between MS and HC – Good test–retest reproducibility – Significantly higher SUV but not VT in HABs vs. MABs	N/A

	Datta et al. (65)	RRMS (16) SPMS (7)	– No association between [^11^C]PBR28 uptake and MRS myo-inositol signal in WM lesions among all patients – Moderate correlation between creatine normalized NAA concentration and [^11^C]PBR28 uptake in WM lesions	– No association between [^11^C]PBR28 uptake and MRS [myo-inositol] in NAWM or GM among all patients – Correlation between higher normalized [myo-inositol] and higher [^11^C]PBR28 binding weighted by WM lesion fraction within patients with high [^11^C]PBR28 binding	– No association between clinical disability and [^11^C]PBR28 binding

	Datta et al. (56)^d^	HC (20) RRMS (17) SPMS (7)	– Heterogeneous patterns of binding in WM lesions – Mean uptake in WM lesions lower when compared to NAWM – Higher proportion of inactive lesions in SPMS vs. RRMS	– Higher uptake in NAWM and thalami in MS vs. HC – Strong positive correlation between median WM lesional and NAWM binding	– Higher proportion of inactive lesions in patients with longer disease duration

	Datta et al. (57)	RRMS (14) SPMS (7)	– [^11^C]PBR28 uptake in WM lesions correlated positively with baseline T2 lesion volume	– [^11^C]PBR28 uptake in NAWM correlated positively with baseline T2 lesion volume – Negative correlation between MTR in NAWM and [^11^C]PBR28 uptake in NAWM in baseline	– Enlarging T2 lesion volumes at 1 year follow-up correlated with higher NAWM and WM lesional [^11^C]PBR28 uptake in baseline in RRMS but not in SPMS – Higher whole brain and GM atrophy rate at 1-year follow-up correlated with higher WM lesional uptake in baseline in SPMS – Non-significant trend for correlation between higher whole brain atrophy rate at 1-year follow-up and higher NAWM uptake in baseline

[^18^F]PBR111	Colasanti et al. (66)	HC (11) RRMS (11)	– Higher uptake in T2 lesional and perilesional WM in RRMS vs. normal WM in HC group	– Non-significant trend for lower whole WM binding in HC vs. RRMS	– Positive correlation between higher lesional-to-nonlesional WM binding ratio and MS severity scores in RRMS

	Colasanti et al. (67)	HC (22) RRMS (11)	N/A	– Higher hippocampal uptake in RRMS vs. HC – No difference in thalamic uptake in RRMS vs. HC	– Positive correlation of higher hippocampal uptake to higher BDI score in RRMS – Higher age associated with higher hippocampal uptake

	Datta et al.^d^ (56)	HC (10) RRMS (10)	– Heterogeneous patterns of binding in WM lesions – No significant difference in lesional vs. NAMW uptake – Higher proportion of inactive lesions in SPMS vs. RRMS	– Higher uptake in NAWM in MS vs. HC – Strong positive correlation between median WM lesional and NAWM binding	– Higher proportion of inactive lesions in patients with longer disease duration

[^18^F]GE180	Vomacka et al. (59)	HC (6) RRMS (17)	– Increased mean uptake in MS lesions	– Higher uptake in WM and thalami in RRMS vs. HC	N/A

[^11^C]FEDAA1106	Takano et al. (58)^a^	HC (5) RRMS (9)	– Reliable lesional binding estimates not obtainable due to noisy time activity curves – High uptake in one Gd+ lesion in one patient	– No difference in global or regional uptake between RRMS and HC	N/A

*TSPO, 18 kDa translocator protein; HC, healthy control; RRMS, relapsing remitting multiple sclerosis; SPMS, secondary progressive multiple sclerosis; PPMS, primary progressive multiple sclerosis; PMS, progressive multiple sclerosis; CIS, clinically isolated syndrome; ROI, region of interest; NAWM, normal-appearing white matter; WM, white matter; Gd+, gadolinium enhancing; Gd−, non-enhancing; EDSS, expanded disability status scale; HAB, high-affinity binder; MAB, mixed-affinity binder; MRS, magnetic resonance spectroscopy; NAA, N-acetyl aspartate; BDI, Beck Depression Inventory; MTR, magnetization transfer ratio*.

*^a^No genotyping for the single nucleotide polymorphism (rs6971) in TSPO gene affecting the radioligand binding affinity, which possibly affects the interpretation of results*.

*^b^Follow-up MRI performed at 1 month after PET imaging for some of the MS patients*.

*^c^Exact disease type not reported; longitudinal PET data reported only for one patient*.

*^d^Study reporting findings from both [^11^C]PBR28 and [^18^F]PBR111, results from both ligands pooled in the lesion binding characterization*.

Despite the advances in genetic testing, other challenges remain in the image analyses and in estimation of the specific binding of these ligands. As with [^11^C]PK11195, some of the specific TSPO binding of second-generation ligands appears to be accounted for by binding to activated astrocytes (68) and endothelial cells (69). In addition, the methodology for individual normalization or the choice of a reference region, presumably free of specific binding, is very varied among the human brain studies using second-generation ligands. For example, use of white matter (60) and caudate nucleus (56, 57) as pseudoreference regions as well as whole brain normalization (70) have been reported but not thoroughly validated for [^11^C]PBR28. In contrast, [^18^F]GE180 appears to have surprisingly low brain uptake in healthy controls (71), which makes the quantification of specific binding even more challenging, although the methodology for total distribution volume estimation appears feasible (72).

## TSPO-PET Imaging Findings in Different Subtypes of MS

### TSPO-PET Imaging in Progressive MS

**Figure 1:**
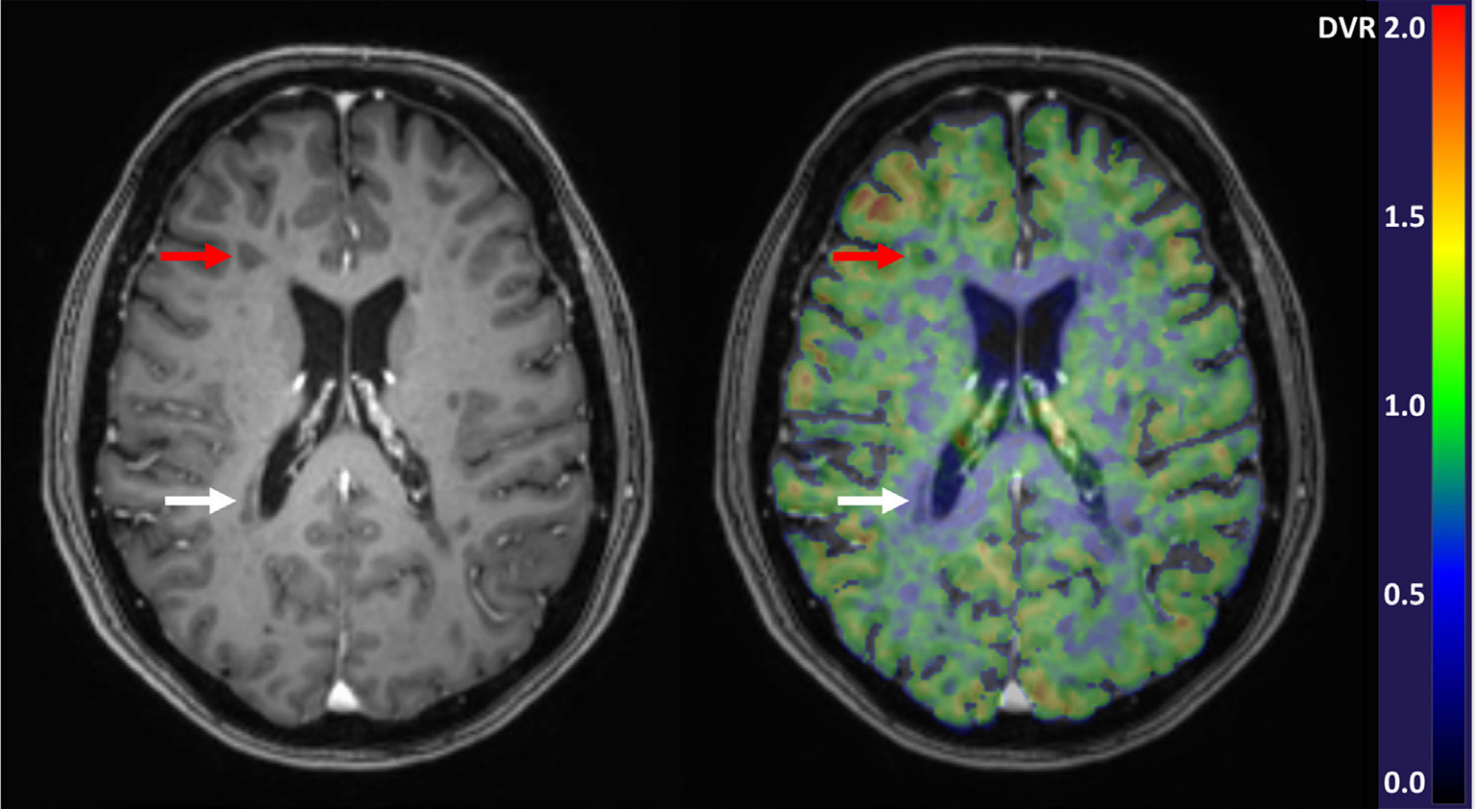
Gadolinium enhanced 3DT1 MRI image (left) and parametric [^11^C]PK11195-PET image overlayed with the 3DT1 image (right). Red arrows point to a chronic active T1-hypointense lesion with increased perilesional [^11^C]PK11195 binding demonstrative of microglial activation, and white arrows point to a chronic inactive lesion with negligible radioligand binding. In the parametric PET image, the color of each voxel represents the intensity of specific radioligand binding measured as distribution volume ratio (DVR) and denoted by the scaled color bar.

Studies of progressive MS have demonstrated an increase in TSPO uptake in the NAWM and NAGM which appears to be related to disease severity and patient age (60). In the NAWM of SPMS patients, the TSPO binding is significantly increased when compared to age-matched healthy controls (20, 30, 46, 47, 60). In PPMS, such studies are still lacking. In addition to quantification of the diffuse microglial activation in the NAWM and NAGM, PET imaging can also be used to differentiate between chronic active (smoldering) and chronic inactive lesions. In particular, the slowly expanding/smoldering lesions are thought to contribute to progression of MS and being able to detect these *in vivo*, and to evaluate the kinetics of the plaque evolution *in vivo*, will likely give new information into the pathology driving the progression. We found that in the brain of advanced SPMS patients, 57% of the plaques were of the chronic active type, with increased TSPO-binding at the plaque edge demonstrating persisting inflammatory activity in these “holes” (20). Figure 1 demonstrates a TSPO-PET image with both chronic active and chronic inactive lesions. Similarly, Giannetti et al. demonstrated heterogeneity in [^11^C]PK11195 binding pattern in black holes (45). Findings from MS studies using later generation TSPO ligands were also in accordance with the above described findings (66, 72).

### TSPO-PET Imaging in RRMS

*In vivo* TSPO-PET imaging has revealed modest microglial activation in the NAWM of RRMS patients, when compared to SPMS (46). Similarly, in neuropathological studies, the diffuse microglial activation outside focal lesions was a feature of progressive disease and was less significant in RRMS patients (7). However, CIS patients who later developed clinically definite MS were shown to have increased TSPO radioligand binding in the NAWM (50). Similarly, during a washout period for a switch in disease modifying therapy, RRMS patients had increased TSPO binding in the NAWM when compared to healthy controls (52). TSPO binding is increased in acute lesions, and T2 lesions have higher TSPO binding during a relapse than during stable disease (39, 48, 53).

## TSPO-PET Imaging as a Prognostic Marker for MS Worsening

Usability of TSPO-PET as a prognostic marker for MS evolution has already been addressed in several studies. Datta et al. found that greater binding of the second-generation TSPO radioligand [^11^C]PBR28 in the NAWM correlated with subsequently greater MRI activity (enlarging T2 lesion volume) among RRMS patients, and with a greater rate of brain volume loss among patients with SPMS (57). This indirectly suggests that the more substantial total inflammatory burden measured using TSPO-PET might predict faster subsequent progression as both enlarging lesions and the brain atrophy rate have prognostic significance for disability progression in MS (73). Another study demonstrated that an adverse clinical outcome in a group of MS patients correlated with increased TSPO binding at baseline (50). Here, a group of patients converting from clinically isolated syndrome (CIS) to clinically definite MS during a follow-up period of 2 years had higher TSPO binding in the NAWM at baseline compared to the group who retained their CIS status (50). Similarly, those SPMS patients whose EDSS improved over a follow-up period of 30 months had lower TSPO-binding in black holes at baseline compared to patients with worsening EDSS (45).

## Effect of MS Therapeutics on Microglial Activation Measured Using TSPO-PET

The greatest potential for TSPO-PET imaging over conventional MRI lies in its ability to detect the diffuse compartmentalized inflammation related to microglial activation, and there are expectations for the usability of PET imaging in the quantification of treatment effects of MS drugs targeting microglial activation. The two published longitudinal TSPO-PET studies evaluating microglial activation in the NAWM of MS are by Ratchford et al. (49), and Sucksdorff et al. (52). In the first study, RRMS patients were evaluated before and after 1 year of glatiramer acetate treatment. The study demonstrated that treatment of RRMS with glatiramer acetate reduced TSPO binding significantly in both cortical GM and cerebral WM when using cerebellum as a reference region. The TSPO-PET study by Sucksdorff et al. included three serial PET images of MS patients. After 6 months of fingolimod treatment no statistically significant reduction in microglial activation could be observed in the NAWM or NAGM in the group of ten individuals taking part in the study. A reduction in microglial activation was observed, however, in T2 lesion areas. Similarly, treatment of a focal lesion in a rat EAE model demonstrated a clear reduction in microglial activation after fingolimod treatment (74). The study by Kaunzner et al. demonstrated reduction in microglial activation in focal inflammatory lesions after natalizumab treatment (53). None of these studies included a prospectively followed MS control group without treatment, and a longitudinal study which would evaluate alteration in microglial activation in untreated MS patients over time is still awaited. In fact, longitudinal TSPO-imaging studies are scarce overall. Kreisl et al. reported recently an increase in TSPO binding among patients with Alzheimer’s disease over a period of 2.4 years, compared to healthy controls (75). Tables 1 and 2 list all known MS studies performed so far using TSPO imaging.

## Future Directions in PET Imaging of Activated Microglia in MS

Despite the established role for TSPO-PET imaging in detecting activated microglia *in vivo* there remain challenges. One is that it is presently not possible to differentiate the anti-inflammatory (M2-type) and pro-inflammatory (M1-type) phenotypes of microglia with TSPO targeting radioligands (76). To date, two radioligands targeting the P2X7 purinergic receptor, namely [^11^C]GSK1482160 (77, 78) and [^18^F]EFB (79), have been developed and tested in animal models of neuroinflammation. Importantly, the expression of P2X7 in microglia has been associated with a pro-inflammatory M1-like phenotype of these cells (80). If further studies with the P2X7-binding radioligands show potential for their use in humans, they could be applied as imaging biomarkers in future longitudinal observational and treatment studies of neuroinflammatory and neurodegenerative conditions.

Several other targets for PET imaging of microglia have also been proposed, including inducible nitric oxide synthase (iNOS), folate receptor β (FRβ), indoleamine 2,3-dioxygenase-1 (IDO-1), kynurenine-3-monooxygenase (KMO), and cannabinoid receptor 2 (CB2) (81, 82). Of these, iNOS and FRβ may have additional value over TSPO, since iNOS is potentially specific for M1-type pro-inflammatory cells, and FRβ for the M2-type homeostatic phenotype of microglia (83, 84). Radioligands for KMO have not yet been developed, and radiotracers for IDO-1 and FRβ have so far been use only in preclinical studies (85–88). Several ligands for CB2 have been developed and tested, but none of these have been found to be suitable for clinical use (82). The first human dosimetry study (89) and one pulmonary imaging study with an endotoxin challenge in healthy subjects using the iNOS-binding radioligand [^18^F]NOS have been reported (90), but no brain imaging studies with this radioligand have been published. However, pitfall of using iNOS-binding radioligands in the estimation of brain microglial activation is that iNOS is expressed also in macrophages and astrocytes, in addition to microglia (91).

## Conclusion

Detection of microglial activation in MS brain using *in vivo* PET imaging has already increased our understanding of MS pathogenesis. In the future, we can expect PET imaging to provide alternative methods to monitor the disease progression, to improve the evaluation of therapeutic needs, particularly in progressive MS, and to help choose MS patients most at risk for progression into therapeutic trials of progressive MS. TSPO-PET could also be used as an important surrogate marker in therapeutic studies of progressive MS. There are still technical challenges, such as the poor signal-to noise ratio of the [^11^C]PK11195 radioligand, and the genetically determined variation in the binding affinity for the second-generation tracers. Moreover, heterogeneity in TSPO image analysis methodology across different imaging centers makes it difficult to perform direct comparisons between the studies. It will be important to harmonize and validate the methodology used in TSPO-PET imaging to allow multi-center studies for evaluation of larger patient cohorts. The great expense and the high technical requirements of nuclear medicine make PET a demanding technology. Nonetheless, the potential of PET imaging to visualize hidden inflammation and other pathogenic determinants in MS brain *in vivo* makes the pursuit of development of yet better ligands a worthwhile effort.

## Author Contributions

Drafting of the manuscript and critical revision of the manuscript: LA, MN, and ER. Study supervision: LA.

## Conflict of Interest Statement

The authors declare that the research was conducted in the absence of any commercial or financial relationships that could be construed as a potential conflict of interest.
